# Early Detection of Adverse Drug Events via 24-h Telephone Services in a Community Pharmacy: A Case Report

**DOI:** 10.1155/crps/6614727

**Published:** 2025-02-18

**Authors:** Masaki Maehara, Masayasu Sugiyama

**Affiliations:** Sugiyama Pharmacy Co., Ltd., 525-2 Mitsusawa, Ogoori, Fukuoka 838-0106, Japan

**Keywords:** 24-h telephone service, aripiprazole, drug–drug, extrapyramidal symptom, lithium

## Abstract

A 57-year-old woman with bipolar disorder (BD) was started on combination therapy with aripiprazole and lithium. At the same time, a community pharmacist administered follow-up through 24-h telephone services for the early detection of adverse events. Four days after starting therapy, the patient called a community pharmacy after working hours and mentioned the occurrence of disabilities, possibly due to adverse effects, including extrapyramidal symptoms (EPSs), to the pharmacist who received the forwarded call. The community pharmacist immediately called the hospital to report the patient's problems and suggested a decrease in doses or withdrawal of the suspected medications to the prescribing doctor. After several hours, the hospital called and informed the pharmacist that the doctor had instructed the patient to discontinue aripiprazole. The pharmacist immediately called the patient, explained the doctor's instructions, and found that the EPS symptoms improved gradually, except for difficulty speaking smoothly. Ultimately, valproic acid was prescribed instead of lithium, resulting in a dramatic improvement in speech difficulties. These results indicate that community pharmacist-administered follow-up and intervention, especially through 24-h telephone services, is crucial for drug safety management, such as early detection of adverse events caused by combination therapy in patients with BD.

## 1. Introduction

Bipolar disorder (BD) is a severe and recurrent chronic mental illness with a worldwide prevalence of more than 1%, leading to various disabilities, including cognitive impairment, inability to participate in social activities, and decreased quality of life (QOL). Furthermore, the mortality rate in patients with untreated BD, including deaths by suicide, is 20 times higher than in the general population [1].

In Japan, combination therapy with second-generation antipsychotics (SGAs), such as aripiprazole, and mood stabilizers, such as lithium, is recommended for the treatment of patients with BD [2]. Aripiprazole is an atypical antipsychotic that acts as a dopamine D2 receptor partial agonist, serotonin 5-HT1A receptor partial agonist, and 5-HT2A receptor antagonist; it is associated with a low risk of extrapyramidal symptoms (EPSs), except for akathisia [3]. The drug also ameliorates dopamine dysregulation, which is recognized as a component of the pathophysiology of mania [4]. Although the exact mechanism of action of lithium is unknown, it is used for the treatment of mania and depression in BD [5].

In contrast, this combination therapy has been shown to cause a wide range of adverse events [6]. In particular, EPS, such as tremors, akathisia, dystonia, tardive dyskinesia, dysphagia, and oculogyric crisis (OGC), are the most common adverse events associated with antipsychotics. In fact, EPS induction has been observed in more than 50% of patients with BD who take SGAs. Compared to SGAs with a lower risk of akathisia, such as quetiapine and olanzapine, aripiprazole appears to be associated with a higher risk of this specific EPS in patients with BD [7]. Furthermore, induction of EPS is known to be a risk factor for a decreased QOL [8], medication withdrawal [9], and initial symptoms of neuroleptic malignant syndrome (NMS) [10]. Moreover, lithium is associated with a risk of toxicity and side effects such as slurred speech, EPS, decreased appetite, tremor, and nausea [5]. Therefore, it is crucial to detect and address these adverse effects early in patients with BD who are receiving SGAs and lithium.

The Japan Pharmaceutical Association promotes community pharmacist-led interventions in clinical settings, with the expectation that this will ensure safe pharmacotherapy and the proper use of pharmaceuticals for local residents and patients [11]. For example, community pharmacists can provide patients with round-the-clock telephone services for queries regarding medications [12]. Consequently, patients can contact community pharmacists even after working hours and receive information on adverse events and drug–drug/food/supplement interactions, among other things. Furthermore, the Japan Pharmaceutical Association has also emphasized the importance of follow-up and officially announced follow-up guidelines (e.g., monitoring medication adherence, medication effectiveness, and adverse events) for patients after the administration of medication [13].

In this case, we demonstrate that a community pharmacist-led follow-up through 24-h telephone services was very useful for early detection and intervention of adverse events in a patient with BD who received combination therapy with lithium and aripiprazole.

## 2. Case Presentation

A 57-year-old woman with depressive symptoms was previously prescribed an antidepressant (escitalopram, 20 mg/day) and hypnotics (brotizolam, 0.25 mg/day; etizolam, 1 mg/day). Subsequently, she experienced manic episodes, including violent language use, and was diagnosed with BD. On day 0, an antipsychotic (aripiprazole, 24 mg/day), mood stabilizer (lithium, 400 mg/day), and hypnotics (etizolam, 1 mg/day; brotizolam, 0.25 mg/day) were prescribed. The pharmacist at our community pharmacy explained the symptoms of adverse effects, such as EPS, caused by these medications and instructed her to call the pharmacy if she experienced these symptoms, even after working hours.

## 3. Pharmacist Follow-Up and Intervention

Four days after starting these medications, she called the community pharmacy that had dispensed her medications after work hours (at 21:00). The pharmacist who received the forwarded call noted that the patient had reported the occurrence of EPS symptoms, such as dysphagia, OGC, and slurred speech (especially with the “L” sounds [La, Li, Lu, Le, and Lo] and “S” sounds [Sa, Si, Su, Se, and So]). Furthermore, she had other symptoms, such as weakness, fatigue, and difficulty moving her limbs, although she did not have fever, which is a main symptom of NMS. Therefore, she was concerned about taking her medications.

The pharmacist suspected that the medication treatment, especially the combination therapy with aripiprazole and lithium, had resulted in the induction of EPS. The pharmacist immediately called the prescribed hospital, described the patient's symptoms to the hospital staff, and suggested a reduction in doses or withdrawal of aripiprazole and/or lithium with the understanding that, if side effects were alleviated by dose reduction or discontinuation, the patient should continue to receive follow-up examinations from the doctor. The pharmacist also noted that if the symptoms worsened, prompt medical attention would be necessary. After a while, the hospital staff, who had contacted the doctor directly, called the pharmacy back to inform them that the prescribing doctor had decided to stop aripiprazole. The pharmacist immediately called the patient and explained the doctor's instructions. The next day (day 5), the pharmacist called the patient and found that her adverse symptoms had gradually improved, except for the difficulty speaking smoothly.

On day 6, the patient visited the pharmacy after a hospital appointment and stated that her adverse symptoms caused by EPS had improved. However, she still had difficulty pronouncing words, which affected her work. On day 7, the pharmacist called her to follow-up on her symptoms and found no improvement in the pronunciation of the “L” and “S” sounds. Therefore, the pharmacist conveyed her symptoms, possibly due to lithium-induced adverse events, and suggested a decrease in lithium doses via a document to the doctor during work hours. After several hours, the doctor called the pharmacy and instructed a decrease in lithium doses from 400 to 200 mg/day. The pharmacist immediately called the patient and explained the doctor's instructions. However, the dose reduction did not improve her speech difficulties. Finally, the doctor decided to prescribe valproic acid (200 mg/day) instead of lithium on day 14. Seven days after the medication change, her pronunciation difficulties had improved drastically, leading to an increase in the valproic acid dose to 400 mg/day. On her visit to the pharmacy on day 49, her manic symptoms had stabilized, and she was satisfied with her improvement in the pronunciation of the “L” and “S” sounds, which no longer caused any issues when speaking with her customers during work.

## 4. Discussion

Combination therapy may exert a synergistic therapeutic effect on the symptoms of BD. However, it may also carry a high risk of adverse events, including EPS. A previous study showed that aripiprazole (as an adjunct to lithium or valproic acid) led to a more significant improvement in manic symptoms than placebo, while the incidence of EPS was higher in the aripiprazole group than in the placebo group (28% vs. 14%) [4]. Furthermore, combination therapy with antipsychotics and lithium has been reported to enhance dopamine receptor blockade, reduce dopamine function, and potentially cause EPS [7, 14]. In addition, the present study showed that EPS developed with combination therapy, and the adverse symptoms of EPS improved after withdrawal from aripiprazole. Taken together, it is always necessary to be cautious of the possible emergence of EPS caused by combination therapy with aripiprazole and lithium in patients with BD.

In fact, the British Association for Psychopharmacology guidelines emphasize the importance of early detection of EPS in patients with schizophrenia. In particular, it should be noted that EPS can lead to lethal NMS [15]. In this case, our patient reported EPS over the phone after starting combination therapy but fortunately did not exhibit symptoms of NMS (e.g., fever, changed mental state, or changes in autonomic symptoms). This highlighted the critical importance of early detection of EPS to prevent progression to NMS [10].

Meanwhile, the present case indicated that the patient's speech problem improved dramatically after switching from lithium to valproic acid. This suggests that the speech impairment was probably induced by lithium. Although the mechanism of these adverse effects is not clear, the treatment guidelines for lithium intoxication describe discontinuing lithium as a sufficient strategy to reduce mild associated toxicity, including slurred speech [16]. Speech impairment due to lithium could significantly affect conversation and, consequently, QOL, thus always requiring careful monitoring and follow-up by community pharmacists.

Previous reports indicated that follow-up and intervention by community pharmacists can improve drug-related problems in patients with mental illness [17–19]. This report is the first to show early detection and management of adverse events, possibly due to the combination therapy of lithium and aripiprazole, achieved through community pharmacist-led follow-up and intervention via telephone services. Specifically, 24-h follow-up and intervention through telephone communication between the pharmacist and the patient or prescribing doctor led to the early detection of adverse events. Subsequent symptoms of adverse effects were completely improved by withdrawing, reducing the dose, or changing suspected medications, according to the pharmacist's suggestion. A limitation of this case was the inability of the pharmacist to communicate directly with the prescribing physician, potentially contributing to the risk of miscommunication. Despite this, the pharmacist ensured that recommendations were clearly conveyed through reliable hospital staff to minimize errors in the present case. Prioritizing direct pharmacist–physician communication in future cases, potentially through telecommunication tools, could further improve the accuracy and effectiveness of interventions. These results strongly suggest that follow-up and intervention by community pharmacists, especially through 24-h telephone services, are highly useful strategies for the early detection of adverse events, such as EPS, which are more likely to develop in patients undergoing combination therapy for BD.

## Data Availability

Research data are not shared due to privacy and ethical considerations.

## References

[1] Grande I., Berk M., Birmaher B., Vieta E. (2016). Bipolar Disorder. *The Lancet*.

[2] Kanba S., Kato T., Terao T., Yamada K., Committee for Treatment Guidelines of Mood Disorders, Japanese Society of Mood Disorders, 2012 (2013). Guideline for Treatment of Bipolar Disorder by the Japanese Society of Mood Disorders, 2012. *Psychiatry and Clinical Neurosciences*.

[3] Pierre J. M. (2005). Extrapyramidal Symptoms With Atypical Antipsychotics: Incidence, Prevention and Management. *Drug Safety*.

[4] de Bartolomeis A., Perugi G. (2012). Combination of Aripiprazole With Mood Stabilizers for the Treatment of Bipolar Disorder: From Acute Mania to Long-Term Maintenance. *Expert Opinion on Pharmacotherapy*.

[5] Grandjean E. M., Aubry J.-M. (2009). Lithium: Updated Human Knowledge Using an Evidence-Based Approach: Part III: Clinical Safety. *CNS Drugs*.

[6] Young S. L., Taylor M., Lawrie S. M. (2015). First do no Harm.“ A Systematic Review of the Prevalence and Management of Antipsychotic Adverse Effects. *Journal of Psychopharmacology*.

[7] Ghaemi S. N., Hsu D. J., Rosenquist K. J., Pardo T. B., Goodwin F. K. (2006). Extrapyramidal Side Effects With Atypical Neuroleptics in Bipolar Disorder. *Progress in Neuro-Psychopharmacology and Biological Psychiatry*.

[8] D’Souza R. S., Hooten W. M., StatPearls (2020). Extrapyramidal Symptoms.

[9] Garcia S., Martinez-Cengotitabengoa M., Lopez-Zurbano S. (2016). Adherence to Antipsychotic Medication in Bipolar Disorder and Schizophrenic Patients: A Systematic Review. *Journal of Clinical Psychopharmacology*.

[10] Woodbury M. M., Woodbury M. A. (1992). Neuroleptic-Induced Catatonia as a Stage in the Progression Toward Neuroleptic Malignant Syndrome. *Journal of the American Academy of Child & Adolescent Psychiatry*.

[11] Yamamoto N. (2022). [The Strategic Plan of the Japan Pharmaceutical Association, the Aspect of a Super Aged Society]. *Yakugaku Zasshi*.

[12] Nakagawa S., Kume N. (2017). Pharmacy Practice in Japan. *The Canadian Journal of Hospital Pharmacy*.

[13] (2022). Guidelines for Patinest Follow-Up During Medication Use (Version 1.2). https://www.nichiyaku.or.jp/assets/uploads/pharmacy-info/followup_1.2.pdf.

[14] Addonizio G., Roth S. D., Stokes P. E., Stoll P. M. (1988). Increased Extrapyramidal Symptoms With Addition of Lithium to Neuroleptics. *The Journal of Nervous and Mental Disease*.

[15] Barnes T. R., Drake R., Paton C. (2020). Evidence-Based Guidelines for the Pharmacological Treatment of Schizophrenia: Updated Recommendations From the British Association for Psychopharmacology. *Journal of Psychopharmacology*.

[16] Gitlin M. (2016). Lithium Side Effects and Toxicity: Prevalence and Management Strategies. *International Journal of Bipolar Disorders*.

[17] Rubio-Valera M., Chen T. F., O’Reilly C. L. (2014). New Roles for Pharmacists in Community Mental Health Care: A Narrative Review. *International Journal of Environmental Research and Public Health*.

[18] Maehara M., Sugiyama M. (2021). A Community Pharmacist’s Intervention in Antipsychotic Drug-Induced Sexual Dysfunction in a Patient With Schizophrenia. *Clinical Case Reports*.

[19] Maehara M., Sugiyama M. (2022). Utilizing Questionnaires for Medication Counselling of Patients Taking Antipsychotics During the COVID-19 Pandemic: A Single Site, Community Pharmacy-Based Survey Study. *Journal of Pharmaceutical Health Care and Sciences*.

